# Cross-sectional study: prevalence of oedema disease *Escherichia coli* (EDEC) in weaned piglets in Germany at pen and farm levels

**DOI:** 10.1186/s40813-023-00343-9

**Published:** 2023-10-26

**Authors:** Pia I. Berger, Steffen Hermanns, Katharina Kerner, Friederike Schmelz, Verena Schüler, Christa Ewers, Rolf Bauerfeind, Marcus G. Doherr

**Affiliations:** 1 Institute of Veterinary Epidemiology and Biostatistics, Freie Universität Berlin, Berlin, Germany; 2 Institute for Hygiene and Infectious Diseases of Animals, Justus Liebig University, Giessen, Germany; 3 IDT Biologika GmbH, Dessau-Roßlau, Germany; 4 Ceva Santé Animale, Libourne, France

**Keywords:** Oedema disease, Piglets, EDEC, Stx2e, F18 fimbriae, Prevalence

## Abstract

**Background:**

*Escherichia coli* bacteria capable of producing the toxin Stx2e and possessing F18-fimbriae (edema disease *E. coli,* EDEC) are considered causative agents of porcine oedema disease. This disease, which usually occurs in piglets shortly after weaning, has a high lethality in affected animals and can lead to high economic losses in piglet rearing. The aim of this cross-sectional field study was to determine the prevalence of EDEC in weaned piglets in Germany at pen and farm levels.

**Results:**

Ninety-nine farms with unknown history of infections with shigatoxin-producing *E. coli* (STEC) and oedema disease were sampled. On each farm, up to five pens were selected for sampling (n = 481). The piglets in these pens were at an age 1–3 weeks after weaning. Single faecal samples (n = 2405) and boot swabs (n = 479) were collected from the floor. On 50 farms, cotton ropes were additionally used to collect oral fluid samples (n = 185) and rope wash out samples (n = 231) from the selected pens. All samples were analyzed by bacterial culture combined with a duplex PCR for the presence of the corresponding genes *stx2e* and *fedA* (major subunit protein of F18 fimbriae). In addition, whole DNA specimens extracted from boot swabs, oral fluid samples, and rope wash out samples were directly examined by duplex PCR for DNA of *stx2e* and *fedA.* A pen was classified as positive if at least one of the samples, regardless of the technique, yielded a positive result in the PCR, and farms were considered positive if at least one pen was classified as positive. Overall, genes *stx2e* and *fedA* were found simultaneously in 24.9% (95% CI 22.1–29.1%) of sampled pens and in 37.4% (95% CI 27.9–47.7%) of sampled farms. Regardless of the presence of F18-fimbriae, *Escherichia coli* encoding for Stx2e (STEC-2e) were found in 35.1% (95% CI 31.0–39.1%) of the pens and 53.5% (95% CI 44.4–63.6%) of the farms sampled.

**Conclusions:**

*Escherichia coli* strains considered capable to cause oedema disease in swine (EDEC) are highly prevalent in the surveyed pig producing farms in Germany. Due to intermittent shedding of EDEC and a potentially low within-farm prevalence, we recommend a combination of different sampling techniques for EDEC monitoring at pen and farm levels. Further studies are needed to understand which STEC-2e strains really pose the risk of causing severe porcine disease.

## Background

Oedema disease (ED) is a worldwide occurring infectious disease of pigs that most often manifests itself during the rearing period of piglets [1]. The disease is caused by certain *Escherichia coli* (*E. coli*) strains that represent a distinct subgroup within the pathotype of Shiga toxin-producing *E. coli* (STEC), designated as oedema disease *E. coli* (EDEC). ED is most frequently observed in the first 2 weeks after weaning, as the associated changes in the microbiome and gut structure make the animals more susceptible to gastrointestinal infections in general, resulting as well in a higher appearance of ED [1]. However, ED may also occur in young fatting pigs. Even wild boars can contract the disease as recent outbreaks in France have demonstrated [2]. After oral uptake, the bacteria multiply in the digestive tract and colonize the small intestine [3]. EDEC are enabled for colonization of the swine intestinal mucosa by adhesive F18 fimbriae. These plasmid-encoded, proteinaceous projections on the bacterial surface mediate attachment of the bacteria to the glycocalyx of porcine enterocytes via specific adhesin-receptor interaction [4–6]. Due to a potent protein cytotoxin, Shiga toxin subtype Stx2e (Stx2e), which is produced by the bacteria and then absorbed into the bloodstream of the pigs, damage occurs to the vessel walls and the subsequent leakage of fluid leads to the formation of oedema in various organs [7–9]. In addition to Stx2e and F18 fimbriae, most EDEC strains produce α-haemolysin and many encode for *E. coli* enterotoxins as well [10–12]. Another adhesion factor, called "adhesin involved in diffuse adherence" (AIDA) could also be isolated from some EDEC strains, however, its importance in the pathogenesis of ED has not been demonstrated yet [13].

A further disease caused by *E. coli*, post weaning diarrhea (PWD), is also common in piglets after weaning and manifests clinically as secretory diarrhea. Most cases of infectious PWD are caused by enterotoxic *E. coli* (ETEC) [14]. PWD and ED can occur concurrently in a farm which may be due to concurrent infections with EDEC and ETEC in one piglet or in one group of piglets. Alternatively, both diseases may be caused in the same piglet by STEC/ETEC hybrids, i.e. adhesive *E. coli* strains that produce Stx2e and enterotoxin(s) simultaneously [15].

Destruction of vascular endothelial cells by Stx2e toxin leads to oedema in various organ systems, revealing the following clinical symptoms: visible oedema in the subcutis of the neck and head, altered vocalizations and dyspnea caused by oedema in the respiratory system, but also ataxia, circling, tremor, rowing movements in lateral position and stupor due to oedema in the central nervous system [9]. Brain oedema is often fatal within few hours of acute disease, which makes therapy attempts ineffective from both animal welfare and economic perspectives [16, 17].

The high economic losses of the disease are caused on the first place by the high lethality (50–90% in acute cases) [4] and on the second place by the often used metaphylaxis with colistin sulfate in the affected group [18]. In addition, experimental demonstration of subclinical ED has supported the hypothesis that subclinical vascular damages are causative in reduced average daily gain in weaned piglets between 8 and 30 kg body weight [19, 20].

For definitive diagnosis of oedema disease certain matrices are examined for EDEC by bacterial culture procedures linked with molecular detection of principal EDEC virulence genes in suspect isolates [1, 14, 21]. Luminal contents of the lower small intestine are most valuable as a diagnostic matrix and may be obtained during necropsy from piglets that succumbed to the disease or from sick piglets that had been euthanized. EDEC can also be isolated from faecal matrices obtained with various techniques: rectal swabs are used to sample individual animals, while pooled faecal samples can be used for testing a group of animals. For isolation of *E. coli* from the animals' environment, boot swabs (using socks) can be collected from the floor of the barn [22]. There are numerous media available for growing *E. coli*, commonly used is sheep blood agar in combination with selective media such as MacConkey, Drigalski, Gassner, and Hektoen agar [14, 21, 23–25]. Since most EDEC strains produce α-haemolysin a haemolytic phenotype on sheep blood agar is often used for presumptive recognition of EDEC colonies [3]. A subsequent PCR assay of individual colonies is a rapid, specific and practical method for the identification of EDEC by their genes encoding for Stx2e and F18 fimbriae [21, 24, 26, 27]. For the detection of F18 fimbriae, the gene of the main protein subunit, called *fedA*, is used.

Although EDEC are considered gastrointestinal pathogens, PCR detection of *stx2e* was also successful with oral fluid samples from piglets [28] as oral fluids had already proven suitable as a matrix for surveillance of various pathogens by both direct and indirect diagnostic approaches [29–31]. To collect oral fluids, pigs are offered a cotton rope for biting and chewing over a defined period before the fluids are harvested from the soaked rope by manual squeezing [29].

Several vaccines for active immunization of piglets and vaccinations of sows and gilts for passive immunization of piglets against ED have been approved in the European Union [32]. Toxoid vaccines produced on the basis of modified Stx2e toxin offer effective protection against the clinical manifestation of ED and thus provide the livestock farmer with the opportunity to reduce economic losses due to treatment costs and/or increased animal deaths [33, 34].

Stx2e-encoding *E. coli* (STEC-2e) have been detected in weaned piglets with ED or PWD worldwide [35–41] but there are almost no studies on general EDEC prevalence in this age group. At least two longitudinal cohort studies in the Midwestern USA revealed that more than 60% of pigs experienced an STEC infection during the fattening period with significant differences in the proportion of STEC positive pigs by site or cohort, respectively. Most STEC isolates of these studies encoded for Stx2e [42, 43]. A seroepidemiologic study in Germany found antibodies against the B-subunit of Stx2e in approximately 53% of the 1,841 sows also suggesting that STEC are remarkably prevalent in commercial pig holdings [44].

Using a cross-sectional study, this work aims to determine the prevalence of EDEC in weaned piglets in Germany. For this epidemiological study, noninvasive methods (single faecal samples from the pen floor, boot swabs, oral fluid and rope wash out samples) were employed to detect Stx2e-encoding *E. coli* (STEC-2e) and EDEC (*E. coli* encoding for both Stx2e and F18 fimbriae) at farm and pen levels.

## Results

### Overview of collected samples

A total of 481 pens with weaned piglets were sampled at 99 farms (mean 4.9 pens/farm). In each pen, five single faecal samples and a boot swab sample (using a pair of socks) were collected from the floor, totalling 2,405 single and 479 boot swab samples. In addition to these samples, cotton ropes were offered to the piglets to collect oral fluids in 231 available pens on 50 farms. A total of 185 oral fluid samples was obtained since not all ropes were sufficiently soaked by chewing piglets during the sampling period (at least 30 min). This eventually resulted in five farms (out of 50) where no oral fluid sample could be obtained. However, a rope wash out sample was harvested from all 231 pens examined.

### Prevalence estimates for STEC-2e and EDEC

STEC-2e or the *stx2e* gene were detected in 35.1% of the examined pens in 53.5% of the farms. In analogy, DNA extracts or putative *E. coli* isolates testing concurrently positive for *stx2e* and *fedA* were collected from 24.9% of the pens and 37.4% of the farms (Table 1). When only those results were considered that had been achieved by the combined testing method of culture and subsequent Stx2e/F18 PCR, STEC-2e were isolated from 121 pens (25.2%) on 47 farms (47.5%) and EDEC from 65 pens (13.6%) on 22 farms (22.2%). Using MALDI-TOF MS all putative *E. coli* isolates that tested positive for *stx2e* or *stx2e* and *fedA* were confirmed as *E. coli* with scores above 2.00.

**Table 1:** Prevalence estimates for STEC-2e and EDEC in weaning piglets in Germany

*Escherichia coli* pathotype	Level	Tested	Positive	Prevalence (%)	95% CI^a^ (%)
STEC-2e	Farm	99	53	53.5	44.4–63.6
STEC-2e	Pen	481	169	35.1	31.0–39.1
EDEC	Farm	99	37	37.4	27.9–47.7
EDEC	Pen	481	120	24.9	22.1–29.1

^a^95% Confidence interval

### Sensitivity of methods for EDEC detection

The methods used for sampling and sample testing in this study were compared with each other for their sensitivities to detect EDEC-positive pens and farms. In this analysis the numbers of positive pens and farms detected with a certain sampling and testing method were related to the number of all EDEC-positive pens and farms of this study that were tested with this method. As depicted in Table 2, each method facilitated detection of EDEC-positive pens, but none succeeded to detect all EDEC-positive pens and farms that were tested with the respective method. The most sensitive approach was to collect rope wash out samples and to examine whole extracted DNA from these samples by Stx2e/F18 PCR. This method facilitated identification of 66.7% of the positive pens, respectively, and 91.3% of the positive farms. A comparison of all samples on which both cultivation and DNA extraction were performed (each with subsequent Stx2e/F18 PCR) showed that the sensitivity of DNA extraction was higher regardless of the sample type used (Table 2). Regarding selectively the examination of samples by bacterial culture and subsequent PCR analysis, single faecal samples was the most sensitive sample type in this study (Table 2).

**Table 2:** Overview of the sensitivity of the samples used in the cross-sectional EDEC study

Sample type	Testing method	Number of pens (EDEC positive)			Number of farms (EDEC positive)		
		Tested (n)	EDEC positive		Tested (n)	EDEC positive	
			N	Percent (%)		N	Percent (%)
Single faecal sample	Culture + PCR	120	63	52.2	37	22	59.5
Boot swab sample	Rinsing + culture + PCR	120	33	27.5	37	17	45.9
	Rinsing + DNA extraction + PCR	120	68	56.7	37	27	73.0
Oral fluid sample	Culture + PCR	66	6	9.1	22	4	18.2
	DNA extraction + PCR	66	34	51.5	22	13	59.1
Rope wash out sample	Rinsing + culture + PCR	78	6	7.7	23	4	17.4
	Rinsing + DNA extraction + PCR	78	52	66.7	23	21	91.3

As a semi-quantitative measure of pen floor contamination by EDEC, prevalence of positive pens was calculated differentiated according to the numbers of positive single faecal samples per pen. As shown in Table 3, pens with one, two, three or five positive faecal samples occurred at rather similar prevalence. In more than 40% of the positive pens EDEC could be isolated from only one or two faecal samples. Table 3 additionally shows the proportion of pens that were classified as EDEC positive by the other methods used in this study. All pens in which five faecal samples proved EDEC positive were also classified as positive by other methods, whereas with only one EDEC positive faecal sample in the pen, only 66.7% of pens were also classified as positive by other methods. In 13.6% of pens, the pen tested positive by other methods while no faecal sample was EDEC positive.

**Table 3:** EDEC positive faecal samples per pen and EDEC status as determined by other approaches

EDEC-positive faecal samples per pen	Number and portion of pens		
	N	EDEC-positive by other sampling and testing method	
		N	Portion (%)
0	418	57	13.6
1	15	10	66.7
2	12	9	75.0
3	15	13	86.7
4	7	6	85.7
5	14	14	100.0
total	481	109	

### Efforts and challenges of the sampling methods

The methods used in this study for non-invasive sampling, sample processing and testing were compared with each other regarding technical problems at pen site and the handling efforts required in the pen and in the laboratory. The on-hand time was recorded as a measure of these efforts. Results of this investigation are summarized in Table 4. A longer time was required for the chewing ropes in the stable, whereas single faecal samples were the most time-consuming sample type in the laboratory.

**Table 4:** Efforts and challenges of sampling methods used in this study

Sample type or sampling device, respectively	Average on-hand time per sample (min)		Number of samples		
	At pen site	In the laboratory (culture + PCR | DNA extraction + PCR)	Total	Technical problems	
				N	Portion (%)
Single faecal sample	1	18 | –	2405	0	0.0
*Pair of PP nonwoven socks*					
Boot swab sample	2	23 | 25	479	0	0.0
*Chewing rope*					
Oral fluid sample	33^a^	18 | 21	450	125	27.8
Rope wash out sample	32^a^	24 | 28			

^a^Including 30 min for surveillance that no accidents happen to the piglets

Collecting faecal samples from the pen floor was performed quickly but required access to the pen. Specific care had to be taken that different fresh droppings were preferably taken for the five single faecal samples. Sampling by boot swabs required no special skills but plastic overboots had to be used consequently with each pair of PP nonwoven socks to avoid cross contamination by the shoe soles. No technical problems appeared during sampling neither with single faecal samples nor with boot swabs (Table 4). Oral fluid and rope wash out samples offered the advantage that pens had not to be entered for sampling thus reducing stress for the piglets. Most animals accepted the cotton ropes as a toy very well and explored them intensively by biting. In this study, a total of 450 chewing ropes were used and sampling caused no issue in 325 of the cases (72.2%). However, no oral fluids could be obtained from 100 ropes (22.2%), which in 37 ropes (8.2%) was because piglets showed no interest in the ropes. In 20 of the used ropes (4.4%) piglets got their ear tags caught what made it necessary to intervene. In 17 of the cases (3.8%) piglets strained the rope so much that it disintegrated, and in 14 ropes (3.1%) the piglets managed to tear ropes completely off their fixation. While 74 ropes experienced only one of these problems, 51 ropes encountered two, three or even four of these issues at the same time. Due to the risk of injury, the piglets were not left unattended during sampling by ropes. The time required for this monitoring was included in the on-hand time presented in Table 4.

## Discussion

This investigation presents the first systematic study on the prevalence of EDEC in weaned piglets in Germany that were not preselected for ED or PWD at the time of sampling. Results of this cross-sectional study indicated prevalence rates of over one-third EDEC-positive and over one-half STEC-2e positive farms, which we consider to be high. High prevalences of STEC-2e have been reported by several studies in the past but those data are hardly to compare as the studies differ greatly in their concepts and methods. While some authors examined only diseased animals [38, 39, 41, 45–48], other studies sampled pigs of differing age groups (fattening pigs or sows) [42–44, 49–51]. The laboratory methods used also varied; in many studies, material obtained during necropsy (small intestinal contents or lymph nodes) was used [38, 39, 46, 52].

We are aware that the accuracy of our prevalence estimates was limited by the numbers of participating farms, of sampled pens, and of samples obtained per pen. Although we found large proportions of tested pens and farms positive for EDEC or STEC-2e, respectively, we suspect that we rather underestimated the prevalence of these bacteria due to a combined effect of possible low within-pen and low within-farm prevalence and the detection limit imposed by our sampling strategy. This assumption is supported by the observation that in 42.9% of the EDEC-positive pens only one or two of five single faecal samples were positive for EDEC, and, similarly, in 18.9% (7 of 37 farms) of the EDEC-positive farms only one of five pens proved positive. It appears reasonable to assume that some EDEC-positive pens and farms remained undetected due to the restraint to only five faecal samples per pen and only five pens per farm.

Considerably higher prevalence was calculated for pens and farms, respectively, being positive for STEC-2e than for those being EDEC-positive since many samples only provided STEC-2e bacteria devoid of the *fedA* gene and whole DNA specimens that proved positive only for *stx2e*. These findings corroborate previous reports by our group and those of others [15, 51]. In a paper from China even 96% of the investigated STEC-2e isolates did not encode for F18 fimbriae [52]. In an earlier study, we confirmed by DNA-DNA hybridization that *fedA* was really missing in STEC-2e isolates that tested *fedA*-negative by PCR (data not published). We therefore assume for the present study that negative PCR results for F18 fimbrial genes were not caused by primer mismatches at the binding sites within target gene *fedA* although this assumption awaits direct experimental proof. In this study we isolated *fedA*-negative STEC-2e from 15.8% of the pens and from 37.4% of the farms. Possibly, we detected those strains more often than other investigators because we did not limit our bacterial culture procedure to blood agar plates and haemolytic colonies but always picked non-haemolytic coliform colonies and coliform colonies from other agar plates as well. The genes for *E. coli* α-haemolysin and F18 fimbriae are located on the same plasmid [53]. Therefore, screening for haemolytic colonies may cause a bias against *fedA*-negative STEC-2e. However, direct evidence is still missing whether *fedA*-negative STEC-2e are virulent and capable to cause ED. As far as we know, there are no data from controlled experimental infection of piglets with F18-negative STEC-2e strains. Genome based phylogeny and comparative genomic analysis may help in future research to assess whether *fedA*-negative STEC-2e represent distinct clonal lineages with so far unknown adhesins or whether they are defective and less virulent descendants from classical EDEC strains.

A primary goal in this study was to determine the prevalence of EDEC in a sufficiently sized sample of weaned piglets in Germany. A representative sampling procedure was not possible because random sampling would require access to population data, which were not available. Nevertheless, we were able to take samples in all regions of Germany with intensive swine production and we detected EDEC and other STEC-2e in all these regions. A history of STEC infection, ED, PWD or any other disease was no criterion to include a farm into or to exclude it from this study. The decisive inclusion criterion was the availability of a sufficient number of piglets at the desired piglet age at the time of the farm visit. However, we cannot rule out the possibility that some farms participated in the study because they struggled with health issues in the nurseries, although we have no evidence of this bias from the farm visit records about symptoms and previous vaccinations (data not shown).

To avoid discomfort or stress to the sampled piglets only non-invasive sampling techniques were applied in this study, although no data were previously available regarding their sensitivity and specificity for detecting these bacterial pathogens. Since none of the techniques succeeded to detect all EDEC positive farms or pens in a pilot study (see materials and methods) a combination of different sample types and sample testing methods was adopted for the present cross-sectional study. Although this approach substantially scaled up the workload for sampling in the barn as well as for sample processing and testing in the laboratory, it increased the sensitivity of the EDEC detection procedure noticeably. In another study investigating the presence of porcine reproductive and respiratory syndrome virus, porcine circovirus type 2 and hepatitis E virus in pig farms, the use of oral fluids, faecal samples, and individual serum samples yielded varying prevalence data [54]. The authors concluded that oral fluids are a low-stress and very efficient sample matrix, and that the viruses studied can be detected with the highest probability. Also in this study, the use of only one examination method would not have detected all positive animal groups.

Each method that was used in our study facilitated detection of EDEC-positive pens but testing whole DNA specimens extracted from rope wash out samples by duplex PCR was the most sensitive procedure. This finding strongly corroborates experimental results published in the only report on this topic that we are aware of [28]. These authors detected the *stx2e* gene by quantitative PCR in oral fluids obtained from pigs in 8 of 18 farms tested. Nonetheless, the frequent positive results with oral fluid and rope wash out samples in this study were surprising as the small and large intestines of pigs are regarded as the natural habitat of EDEC and other *E. coli* pathotypes but not the oral cavity [1]. We observed repeatedly that cotton ropes touched the dirty skin of piglets while being explored and we noticed faecal staining on many ropes upon receipt in the laboratory. We therefore assume that EDEC and DNA of genes *stx2e* and *fedA* recovered from the cotton matrix rather originated from faeces or faecal EDEC, respectively, than from oral fluids. Alternatively, EDEC could have been transiently present on the oral mucosa because the respective piglet may have orally explored faecal matter or had ingested feed or water contaminated with faeces shortly before. Coprophagy is a normal behavior known from piglets although performed to a lesser degree than by rodents and lagomorphs [55]. That natural behavior is probably the reason why *E. coli* can be found in large numbers in tonsils of healthy pigs at slaughter [56, 57]. Nevertheless, it remains to be investigated whether the oral or laryngeal mucosa or the tonsils of swine are indeed natural colonization sites of STEC-2e that have remained unrecognized so far.

Based on our results that were achieved under field conditions, we rate the described sampling with cotton ropes as a valuable and less laborious technique for non-invasive group-level sampling from weaned piglets to monitor *E. coli* pathotypes in this age cohort by PCR. On the other hand, rope sampling was the only method in this study that repeatedly caused problems, such as reluctancy of piglets, demolished ropes, ropes teared into single strings, ropes teared to the pen ground, single rope strings entangled with ear tags or the risk of detached strings to be swallowed. Therefore, we would like to recommend that piglets should be observed carefully while ropes are exposed, and action should be taken immediately if piglets are in danger to get injured. Additionally, some cotton ropes were not soaked abundantly with oral fluids, so that no fluid or no sufficient volume was recovered even by intense squeezing or handwringing as suggested by others [58]. No or poor yield was possibly due to reluctance or timidity of some piglets. Training of piglets prior to sampling or flavoring the rope matrix with sugar solution could have accustomed and motivated the animals [59], however, those steps were beyond the scope of the project. To tackle the problem of poor yield, we rinsed each rope with PBS and treated the resulting PBS suspension as a separate sample (rope wash out sample) in addition to the oral fluid sample harvested by simple wringing. Clearly, the rinsing procedure increased the workload significantly, but it paid off as 5 of 46 single ropes and pair of ropes proved positive for DNA of *stx2e* and *fedA* or for EDEC bacteria that otherwise would not have been examined at all. Furthermore, positive rates were considerably higher for rope wash out samples than for oral fluid samples. However, we could not do without oral fluid samples since some cotton ropes proved positive in this sample type but negative in the rope wash out sample. Consequently, we would recommend our extensive rope processing procedure for similar investigations in the future.

## Conclusions

Our findings suggest that Stx2e-encoding *E. coli* are common in German pig farms and that weaned piglets are frequently exposed to EDEC and other STEC-2e. Whether the high prevalence estimates correlated with the occurrence of clinical signs of ED, and possible economic losses, however, was not assessed in this study. In any case, pig holders should be aware of the threat and specifically address ED in health and welfare monitoring. Controlling possible risk factors that may promote the infection with EDEC appears crucial to maintain pig health at nurseries and to prevent costly outbreaks. Due to intermittent shedding of EDEC and due to a potentially low within-farm prevalence, we recommend a combination of different sampling techniques for EDEC monitoring. Chewing rope sampling is a novel, valuable and less laborious technique in this respect but specific precautions and prearrangements must be considered to make it highly efficient and to protect piglets from injuries.

## Methods

### Study design

Samples were collected in a cross-sectional study with non-invasive methods from weaning piglets at pen level in pig farms in Germany. Given the limitations in time and personnel both at the level of farm visits but even more at the level of the laboratory responsible for testing pen-level samples from all sampling protocols and in different tests, it was decided to limit the number of farms to be recruited for the study to 100. This resulted in a precision in the prevalence estimate of approximately ± 10% at farm level and ± 5% at pen level, which was considered sufficient to achieve the overall study objective. During one-time visits samples had to be collected from five randomly selected pens per farm. Four sample types were collected concurrently in each of these pens: (a) five single faecal samples from the pen floor, (b) a boot swab sample from the pen floor, (c) an oral fluid sample, and (d) a rope wash out sample. All samples were examined systematically for putative *E. coli* bacteria by culture methods. Aliquots of boot swab samples, oral fluid samples, and rope wash out samples were also submitted to whole DNA extraction. Subsequently, putative *E. coli* isolates and extracted whole DNA specimens were tested by polymerase chain reaction (PCR) for carriage or presence of genes *stx2e* and *fedA*, respectively.

### Bacterial reference strains

*Escherichia coli* strains E57 and HB101 were used as PCR positive and negative controls, respectively. Strain E57 (Stx2e, ST-Ia, ST-II, F18 fimbriae) had been kindly provided by C. Wray, Central Veterinary Laboratory, Weybridge, UK [60]. Strain HB101 (*E. coli* K12- and B-derived strain) did not encode any of the relevant virulence factors and had been purchased from the German Collection of Microorganisms and Cell Cultures GmbH, Braunschweig, Germany. For PCR analysis reference strains were aerobically grown in 2 mL of lysogeny broth (37 °C, 18–20 h).

### Pig farms

Ninety-nine pig farms in Germany were recruited for the project through their herd veterinarian. Participation in the project was voluntary and farms were not reimbursed; however, they received the results of the laboratory tests free of charge. Inclusion criteria included a reasonable size to ensure that the farms—at the time of the visit—had at least 5 pens with piglets 1–3 weeks after weaning to be sampled. Additional effort was made to achieve a reasonable geographic coverage of the main swine producing regions in Germany. Prior to the visit, the farms were informed about the project objectives and the process of the farm visit. Participating farms kept a mean of 568 sows (median 240, range 0–10,800) and a mean of 2472 weaned piglets (median 1180, range 150–34,000). The average weaning age of the sampled piglets was 25.6 days of life.

### Sampling protocol at farm level

In advance of the cross-sectional study, a pilot study was conducted to optimize the methodology of sampling, sample processing and sample testing in terms of practicability as well as specificity and sensitivity of the STEC strain detection [22]. In the subsequent field study, fifty of the participating farms were visited by the project veterinarian, the other samples were collected from forty-nine farms by the herd veterinarians. These were familiar to the sampling methods before the farm visit and received illustrated and verbal instructions on sample collection and ready-prepared kits with labelled materials. Every farm was visited once for sampling from December 2018 through March 2020. On every farm, up to five pens (if available) with piglets at days 8 through 21 after weaning were randomly selected for sampling. Five single faecal samples were collected at different locations from the floor of each pen. If possible, fresh faeces was sampled which could be clearly assigned to a piglet. An additional faecal sample was collected using a pair of disposable polypropylene (PP) nonwoven socks (Hygostar Überschuhe Med, Franz Mensch GmbH, Buchloe, Germany) as boot swabs in each pen. For sampling a pen, new polyethylene overboots (Hygostar Überstiefel PE, Franz Mensch GmbH, Buchloe, Germany) were pulled over the boots and then covered with the socks before all areas of the pen were walked systematically to gather a representative sample. A pair of socks was treated as one boot swab sample. On the 50 farms visited by the project veterinarian, oral fluid samples and rope wash out samples were also collected in the selected pens by offering the piglets a single cotton rope (up to 25 piglets in the pen) or two ropes (larger pens) for playful chewing. The unbleached cotton ropes (Swine Oral Fluid Collection Kit, IVD Gesellschaft für Innovative Veterinärdiagnostik mbH, Seelze-Letter, Germany) were suspended at places in the pen that were easily accessible by the piglets and not near food or water dispensers. Using two cable ties each, one rope end was fastened to the pen wall so that the lower end was at animals' shoulder hight level. Ropes were left for chewing for 30 min, then removed, and oral fluids were harvested from each rope immediately after removal by intense manual compression. All samples and ropes were boxed and shipped to the laboratory at the Institute of Hygiene and Infectious Diseases of Animals, Giessen, Germany, for further processing. Frozen cooling packs were added to each package to keep shipments below 10 °C. Poblems encountered during the sampling process were documented at the pen level.

### Sample processing

Approximately 90% of the shipped samples were treated immediately upon arrival at the laboratory. Others were stored at 4 °C for up to 65 h until further processing. Each pair of PP nonwoven socks was transferred into a 1 L-Erlenmeyer flask containing 300 mL of 1 × PBS. Similarly, each cotton rope was removed from the bag and transferred into a 2 L-beaker containing 200 mL of 1 × PBS.

Subsequently, flasks were agitated horizontally at 4 °C for 18 ± 2 h on an orbital shaker (90 rpm). Then, 50 mL of PBS suspension was harvested from each flask and stored in screw-top tubes under the names boot swab sample and rope wash out sample, respectively. When two ropes were available from a pen both ropes were processed individually but PBS suspensions obtained were pooled at a ratio of 1:1 and treated as one rope wash out sample. Similarly, the oral fluids harvested from these two ropes were pooled and treated as one oral fluid sample.

### Isolation of putative *E. coli* colonies

Material from each sample was transferred and streaked for single bacterial colonies on a set of solid media consisting of a sheep blood agar plate, a Gassner agar plate, and a RAPID´E.coli 2/agar plate (Bio-Rad Laboratories, Hercules, CA, USA). Cultures were incubated for approx. 20 h, at 37 °C (sheep blood and Gassner agar plates) or 43 °C (RAPID´E.coli 2/agar plates). Subsequently, 8 putative *E. coli* colonies were picked per sample and stored individually as pure bacterial suspensions in lysogeny broth for further analysis. A single colony was regarded as putative *E. coli* according to the following criteria: (a) circular, shiny, greyish, diameter of 1.0–2.0 mm on sheep blood agar, (b) deep blue with a blue halo, diameter of 1.0–2.5 mm on Gassner agar, (c) violet to pink colony, diameter of 0.5–1.5 mm on RAPID´E.coli 2/agar plates. If haemolytic and non-haemolytic putative *E. coli* colonies occurred on the same sheep blood agar plate representative colonies of both phenotypes were picked.

### DNA extraction

Whole DNA was extracted from aliquots of each boot swab sample as well as each oral fluid and rope wash out sample. The applied method was essentially based on the procedures of Jones et al*.* [61] and Dünser et al*.* [62]. Briefly, a 2 mL-aliquot of the PBS suspension was centrifuged (16,000 × *g*, 2 min). The pellet was resuspended in 200 µL of 1 × PBS, mixed with 1 ml of lysis buffer [6 M guanidine thiocyanate, 22 mM EDTA, 0.1 M Tris HCl (pH 6.4), 0.65% Triton X-100], and incubated (room temperature (RT), 1 h). Then, the mixture was centrifuged (16,000 × *g*, 1 min). The supernatant was harvested, mixed with 50 µL of DE suspension [20% (w/v) diatomaceous earth in 0.17 M HCl], and incubated (RT, 10 min). The suspension was agitated thoroughly before it was centrifuged (16,000 × *g*, 1 min). The residual pellet was washed twice with 2 × 200 µL of washing buffer [6 M guanidine thiocyanate, 0.1 M Tris HCl (pH 6.4)], twice with 2 × 200 µL of ice-cold 70% ethanol and once with 200 µL of acetone. With each wash the pellet was vortexed until it was thoroughly dispersed, and the resulting suspension was centrifuged (16,000 × *g*, 1 min). The acetone pellet was air dried (56 °C, 15 min) und subsequently dissolved in 75 µL of storage buffer [10 mM Tris HCl (pH 8.4), 1.0 mM EDTA]. The resulting solution was cleared by centrifugation (16,000 × *g*, 1 min) and stored at – 20 °C until further use.

### Stx2e/F18 PCR

Putative *E. coli* isolates and extracted whole DNA specimens were tested for DNA of genes *stx2e* and *fedA* with an in-house duplex PCR as described previously [21]. This assay synergized primers Stx2e-F1 and Stx2e-F2 of the *stx2e* detection PCR of Scheutz et al. [63] with primers F18-1 and F18-2 designed by Casey & Bosworth [27] for *fedA* detection, the gene of the major F18 fimbrial subunit FedA [27]. Stock solutions of each primer (Eurofins Genomics, Ebersberg, Germany) had been adjusted to 100 µM with sterile ddH_2_O. The primer mix was composed of 800 µL of sterile ddH_2_O and 50 µL of each primer stock solution. Each PCR reaction mix contained 19.8 µL of sterile ddH_2_O, 3 µL of DreamTaq buffer, 3 µL of duplex primer mix, 1 µL of dNTP stock solution (4 mM each dNTP), 0.2 µL of Thermo Scientific™ DreamTaq DNA polymerase solution (Life Technologies GmbH, Darmstadt, Germany). Finally, 3 µL of the respective specimen (bacterial culture in lysogeny broth or extracted whole DNA specimen) or 3 µL of ddH_2_O (in case of reagent negative control), respectively, was added. DNA amplification was accomplished on a thermal cycler (T Professional Trio 48, Biometra GmbH/Analytik Jena AG, Göttingen, Germany) using the following program: 1 initial cycle at 94 °C for 5 min; 40 cycles at 94 °C for 30 s, 56 °C for 30 s and 72 °C for 60 s; 1 final cycle at 72 °C for 60 s. Subsequently, all mixes were cooled and stored at − 20 °C. PCR products were analysed by horizontal agarose gel electrophoresis as described elsewhere [21]. Amplicons generated from target genes had sizes of 411 bp (*stx2e*) and 313 bp (*fedA*), respectively.

### MALDI-TOF mass spectrometry

All putative *E. coli* isolates that tested PCR-positive for *stx2e* were assessed for their species assignment by matrix-assisted laser desorption/ionization time-of-flight mass spectrometry (MALDI-TOF MS) on a Microflex LT mass spectrometer system (V3.3.1.0, Bruker Daltonics, Bremen, Germany). MALDI-TOF MS was performed according to manufacturer’s instructions. The spectra obtained were compared against reference spectra of a commercial MALDI Biotyper database (MBT 7854 MSP library; Bruker Daltonics). A score value ≥ 2.0 indicated species identification; a score value of 1.7 up to 2.0 indicated genus identification, and a score value < 1.7 indicated no identification.

### Statistical analyses

The laboratory results were assembled and processed in MS Excel. Outcome variables were created to summarize the individual sample results for STEC-2e, EDEC, *stx2e*, and *stx2e*/*fedA* status at the pen and farm level. This was done for all sample types, i.e., single faecal samples, boot swab samples, oral fluid and rope wash out samples. A sample was considered positive for STEC-2e when Stx2e-encoding *E. coli* was isolated or *stx2e* was detected by PCR in the DNA extracted from that sample. In analogy to this, a sample was considered positive for EDEC when *E. coli* encoding both for Stx2e and F18 fimbriae was isolated or when *stx2e* and *fedA* were simultaneously detected in the DNA extracted from that sample. Pens were considered positive when at least one of the respective samples was positive for the analytical target. Farms were considered positive when at least one pen was classified as positive. The positive classification of samples, pens and farms was done assuming that the specificity of the test system (DNA detection using PCR) was 100%. A distinction was also made between (a) finding of STEC-2e or EDEC by bacterial culture, (b) finding of *stx2e* or *stx2e*/*fedA* by PCR in DNA extracts, and (c) a complete evaluation of all samples taken, and all laboratory techniques applied. Statistical prevalence was assessed using the software packages SPSS v25 and STATA v15. Frequencies with 95% confidence intervals were generated for all previously created sample classification variables.

## Data Availability

The datasets used and/or analysed during the current study are available from the corresponding author on reasonable request.

## Abbreviations

AIDA: Adhesin involved in diffuse adherence, an adhesion factor of bacteria
CI: Confidence interval
DNA: Deoxyribonucleic acid
*E. coli*: *Escherichia coli*
ED: Oedema disease
EDEC: Stx2e- and F18 fimbriae-encoding *E. coli* (*E. coli* carrying the genes *stx2e* and *fedA*)
ETEC: Enterotoxic *E. coli*
F18 fimbriae: Proteinaceous projections on the bacterial surface called “F18”
*fedA*: Gene encoding for the major protein subunit of F18 fimbriae
hrs: Hours
min: Minutes
PBS: Phosphate-buffered saline
PCR: Polymerase chain reaction
PP: Polypropylene
PWD: Post weaning diarrhea
STEC: Shiga toxin-encoding/-producing *E. coli*
STEC-2e: *E. coli* encoding for Shiga toxin subtype Stx2e *(E. coli* carrying the gene *stx2e* irrespectively whether they also carry other virulence genes such as *fedA)*
Stx2e: Shiga toxin subtype Stx2e
*stx2e*: Gene encoding for the Shiga toxin subtype Stx2e
